# Eosinophils promote CD8^+^ T cell memory generation to potentiate anti-bacterial immunity

**DOI:** 10.1038/s41392-024-01752-0

**Published:** 2024-02-28

**Authors:** Jun Zhou, Jiaqi Liu, Bingjing Wang, Nan Li, Juan Liu, Yanmei Han, Xuetao Cao

**Affiliations:** 1 Institute of Immunology, Zhejiang University School of Medicine, Hangzhou, 310058 China; 2 National Key Laboratory of Immunity and Inflammation, Institute of Immunology, Naval Medical University, Shanghai, 200433 China; 3 Department of Immunology, Center for Immunotherapy, Institute of Basic Medical Sciences, Peking Union Medical College, Chinese Academy of Medical Sciences, Beijing, 100005 China; 4 Institute of Immunology, College of Life Sciences, Nankai University, Tianjin, 300071 China

**Keywords:** Infection, Adaptive immunity

## Abstract

Memory CD8^+^ T cell generation is crucial for pathogen elimination and effective vaccination against infection. The cellular and molecular circuitry that underlies the generation of memory CD8^+^ T cells remains elusive. Eosinophils can modulate inflammatory allergic responses and interact with lymphocytes to regulate their functions in immune defense. Here we report that eosinophils are required for the generation of memory CD8^+^ T cells by inhibiting CD8^+^ T cell apoptosis. Eosinophil-deficient mice display significantly impaired memory CD8^+^ T cell response and weakened resistance against *Listeria monocytogenes* (*L.m*.) infection. Mechanistically, eosinophils secrete interleukin-4 (IL-4) to inhibit JNK/Caspase-3 dependent apoptosis of CD8^+^ T cells upon *L.m*. infection in vitro. Furthermore, active eosinophils are recruited into the spleen and secrete more IL-4 to suppress CD8^+^ T cell apoptosis during early stage of *L.m*. infection in vivo. Adoptive transfer of wild-type (WT) eosinophils but not IL-4-deficient eosinophils into eosinophil-deficient mice could rescue the impaired CD8^+^ T cell memory responses. Together, our findings suggest that eosinophil-derived IL-4 promotes the generation of CD8^+^ T cell memory and enhances immune defense against *L.m*. infection. Our study reveals a new adjuvant role of eosinophils in memory T cell generation and provides clues for enhancing the vaccine potency via targeting eosinophils and related cytokines.

## Introduction

CD8^+^ T cells are essential for mediating host protection against pathogenic microorganisms and tumors via exerting cytotoxic effector functions.^1–3^ During acute infection, a typical CD8^+^ T cell response is usually categorized into three phases: expansion, contraction, and memory generation.^4,5^ Survival of CD8^+^ T cells during early stage of infection is crucial for generating long-term memory response and effective immune defense.^6,7^ Dysfunction of CD8^+^ T cell memory has been closely linked to chronic infection, autoimmune diseases, and cancer.^8–10^ A more comprehensive understanding of the mechanisms for generating cytotoxicity and long-term memory of CD8^+^ T cells may reveal new strategies to motivate the adaptive immune response to eliminate pathogenic microorganisms and tumor.

The communications between CD8^+^ T cells and other immune cell subsets were increasingly shown to modulate the generation of diverse populations of memory CD8^+^ T cells. CD4^+^ T cells could facilitate CD8^+^ T cell memory by modulating metabolic pathways at initial priming.^11^ Tissue-resident CD4^+^ T cells also promote the generation of CD8^+^ T cell memory following the resolution of infection.^12^ Notably, various kinds of innate immune cells are widely increased or activated during infection and inflammation and carry out variable functions in shaping host defensive immunity. One recent study shows that alveolar macrophages could enhance the lung-resident CD8^+^ T cell memory.^13^ It is important to further reveal the precise contributions of innate immune cells in the establishment of immunological memory.

Eosinophils are cytotoxic effector cells with crucial roles in host inflammatory responses to helminth infection and allergic diseases.^14–17^ Eosinophilic inflammation is implicated in multiple pathogenesis including asthma, pathogenic infections such as COVID-19, hyper-eosinophilic syndromes, and tumors.^18–20^ Recent studies report that eosinophils interact with various players of inflammatory response including lymphocytes to regulate their functions.^21,22^ Eosinophils were shown to exacerbate the pathogenesis of IL-23-Th17 cell-induced colitis and promote IgA-producing plasma cell for the maintenance of gut immune homeostasis.^23,24^ One recent study using single-cell profiling identified the recruitment of eosinophils, monocytes and Th2 cells in eosinophilic chronic rhinosinusitis.^25^ However, the interplay between eosinophils and CD8^+^ T cells in the infection and immunity still remains elusive.

In this study, we utilized the OVA-expressing *Listeria monocytogenes* (*L.m*.-OVA) to infect eosinophil-deficient ∆dblGATA-1 mice and analyzed the CD8^+^ T cell memory. We demonstrate that eosinophil deficiency causes impaired memory generation and weakened immune defense against *L.m*. infection. The eosinophils secrete IL-4 to inhibit *L.m*. infection-induced apoptosis of CD8^+^ T cells to promote immunological memory and subsequent protection against bacterial infection. Our findings outline new clues for modulating eosinophil networks to facilitate vaccine efficacy via augmentation of T cell memory.

## Results

### Eosinophils deficiency impairs memory CD8^+^ T cell generation

**Fig. 1:**
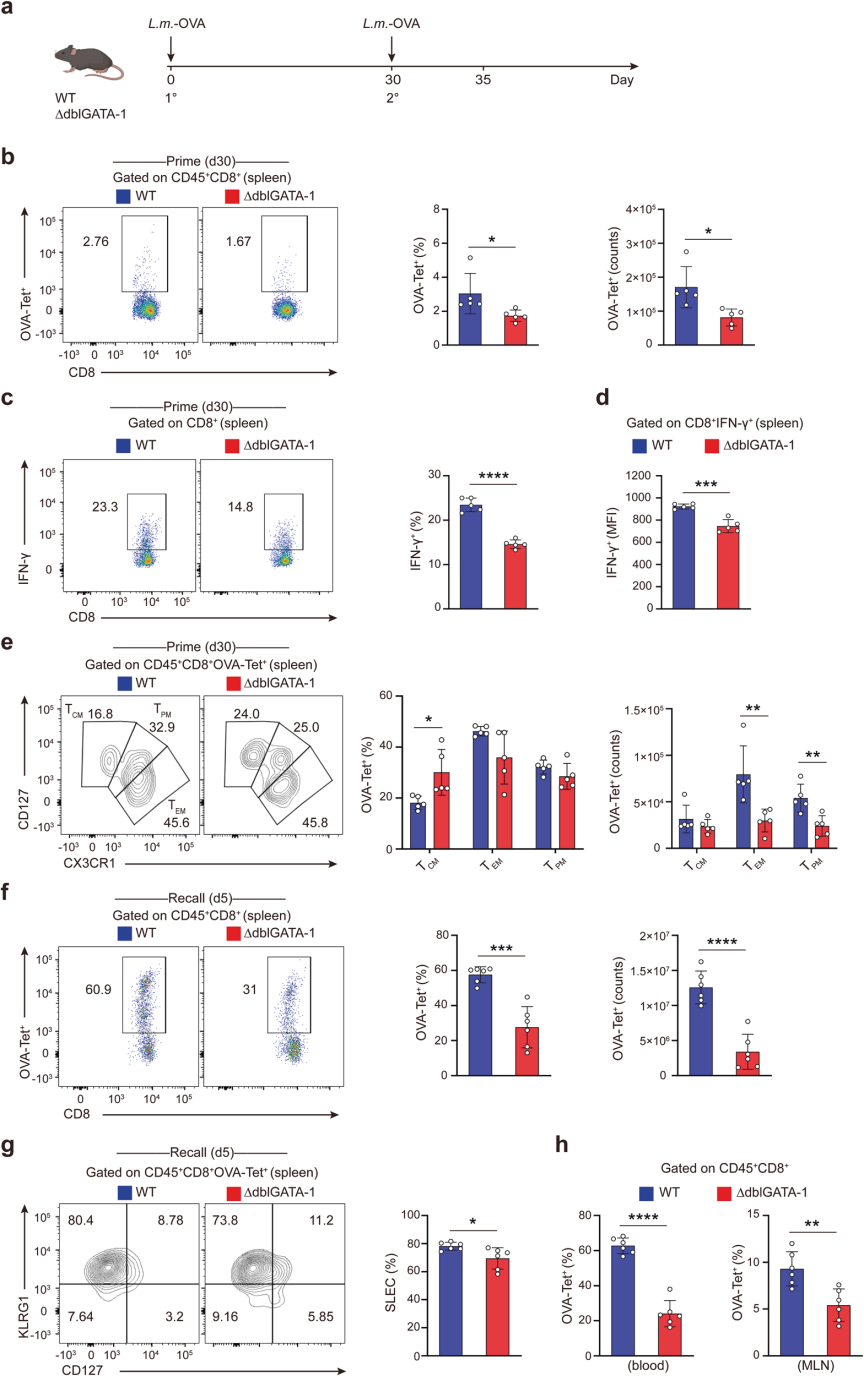
Eosinophils deficiency impairs memory CD8^+^ T cell generation. **a** Diagram of the mouse primary and secondary infection model by i.p. injection of *L.m*.-OVA to wild-type (WT) and eosinophil-deficient (∆dblGATA-1) mice. **b** Representative dot plots of FACS analysis and quantification of percentages and counts of splenic OVA-Tet^+^CD8^+^ T cells in WT and ∆dblGATA-1 mice on d30 after *L.m*.-OVA infection. **c**, **d** Representative dot plots of FACS analysis of intracellular IFN-γ staining of splenic CD8^+^T cells in WT and ∆dblGATA-1 mice and quantification of the percentages (**c**) and IFN-γ mean fluorescence intensity (MFI) (**d**) of IFN-γ^+^CD8^+^ T cells on d30 after *L.m*.-OVA infection. **e** Representative dot plots and statistical analysis of splenic memory CD8^+^T cell subsets from WT and ∆dblGATA-1 OVA-Tet^+^CD8^+^ T cells on d30 after *L.m*.-OVA infection. **f** Representative dot plots of FACS analysis and quantification of percentages and counts of splenic OVA-Tet^+^CD8^+^ T cells in WT and ∆dblGATA-1 mice on d5 after secondary infection (recall). **g** Representative dot plots of FACS analysis and quantification of percentages of splenic OVA-specific SLECs in WT and ∆dblGATA-1 mice after recall response. **h** Quantification of percentages of OVA-Tet^+^CD8^+^ T cells in the blood and MLNs of WT and ∆dblGATA-1 mice after recall response. Data are mean ± SD of one representative experiment. Similar results were seen in two independent experiments with *n* = 5–6 mice per group. Unpaired Student’s t tests. *p < 0.05, **p < 0.01, ***p < 0.001, ****p < 0.0001

To figure out the influence of eosinophils on CD8^+^ T cell fate and response, we utilized ∆dblGATA-1 mice that completely lack eosinophils^26^ and established primary and secondary infection model by intraperitoneal (i.p.) injection of *L.m*.-OVA to WT and ∆dblGATA-1 mice (Fig. 1a). Flow cytometry analysis confirmed the efficient elimination of splenic eosinophils in ∆dblGATA-1 mice (Supplementary Fig. 1a). There were no differences in CD4^+^ T cells, CD8^+^ T cells, CD19^+^ B cells and CD11c^+^ DCs between WT and ∆dblGATA-1 mice, indicating that deficiency of eosinophils does not influence the development of T cells, B cells or DCs (Supplementary Fig. 1b–d). Interestingly, ∆dblGATA-1 mice showed decreased proportions and counts of antigen-specific CD8^+^ memory T cells [OVA-tetramer (Tet)^+^CD8^+^ T cells] in the spleens as compared to WT mice on d30 post infection (p.i.) (Fig. 1b). Besides, ∆dblGATA-1 mice displayed reduced expression and mean fluorescent intensity (MFI) of IFN-γ in splenic CD8^+^ T cells on d30 p.i. (Fig. 1c, d). The proportions of antigen-specific CD8^+^ memory T cells in the mesenteric lymph nodes (MLNs) between ∆dblGATA-1 and WT mice on d30 showed no difference (Supplementary Fig. 1e). We next analyzed the expression of CD127 and CX3CR1 to figure out effector memory (T_EM_), central memory (T_CM_) and peripheral memory (T_PM_) CD8^+^ T cell subsets.^27,28^ The counts of these memory populations were decreased in ∆dblGATA-1 mice compared to WT mice (Fig. 1e). Therefore, eosinophils promote the generation of memory CD8^+^ T cells at d30 post infection.

Then we challenged WT and ∆dblGATA-1 mice with *L.m*.-OVA on d30 after primary infection (recall). We observed impaired generation of splenic OVA-specific CD8^+^ T cells in ∆dblGATA-1 mice (Fig. 1f). The short-lived effector T cells (SLECs, KLRG1^high^CD127^low^) were decreased in ∆dblGATA-1 mice (Fig. 1g). Consistently, ∆dblGATA-1 mice showed decreased OVA-specific CD8^+^ T cells in the blood and MLNs on d5 after secondary infection (Fig. 1h). CD8^+^ T cells were mainly located in the T cell area of the white pulp (WP) on d30 after primary infection, and migrated to the red pulp (RP) where eosinophils were mainly located on d5 after secondary infection (Supplementary Fig. 1f), suggesting an interaction between eosinophils and CD8^+^ T cells in the red pulp of spleen post infection. Thus, eosinophils were required for the generation of memory CD8^+^ T cells in vivo.

### Eosinophils deficiency weakens CD8^+^ T cell response upon infection

**Fig. 2:**
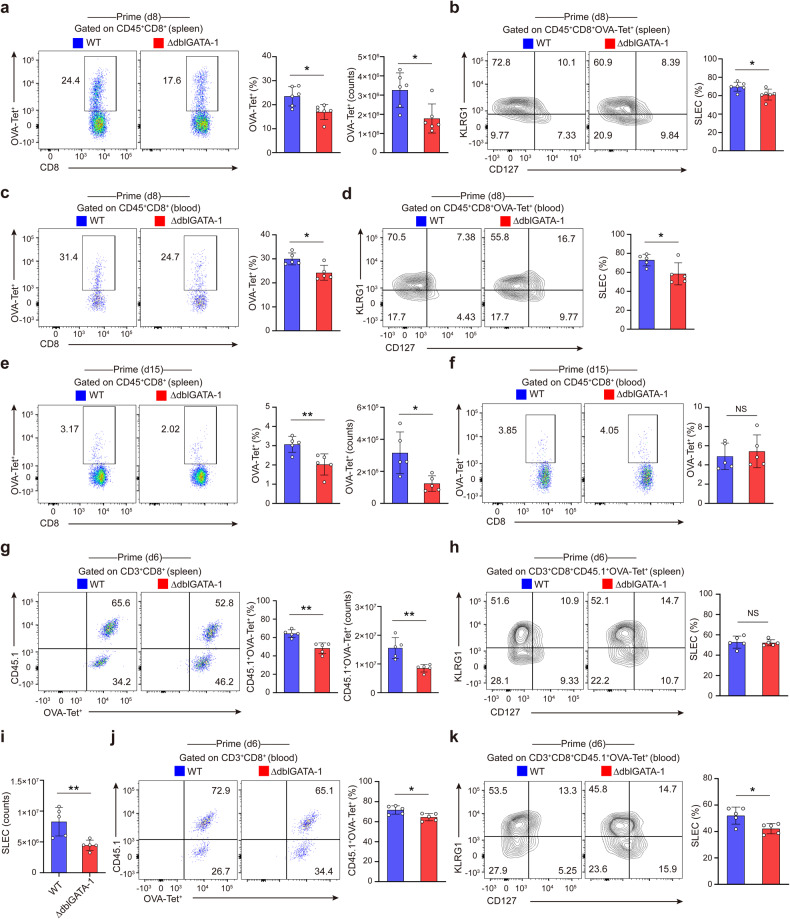
Eosinophils deficiency weakens CD8^+^ T cell response upon infection. **a**, **b** Representative dot plots of FACS and statistical analysis of splenic OVA-Tet^+^CD8^+^ T cells (**a**) and OVA-specific SLECs (**b**) of WT and ∆dblGATA-1 mice on d8 after *L.m*.-OVA infection. **c**, **d** Representative dot plots of FACS and statistical analysis of OVA-Tet^+^CD8^+^ T cells (**c**) and OVA-specific SLECs (**d**) in the blood of WT and ∆dblGATA-1 mice on d8 after *L.m*.-OVA infection. **e, f** Representative dot plots of FACS and statistical analysis of OVA-Tet^+^CD8^+^ T cells and in the spleens (**e**) and blood (**f**) of WT and ∆dblGATA-1 mice on d15 after *L.m*.-OVA infection. **g**–**i** Representative dot plots of FACS and statistical analysis of splenic CD45.1^+^OVA-Tet^+^CD8^+^ T cells (**g**) and SLECs (**h**, **i**) of WT and ∆dblGATA-1 mice on d6 after *L.m*.-OVA infection. **j, k** Representative dot plots of FACS and statistical analysis of CD45.1^+^OVA-Tet^+^CD8^+^ T cells (**j**) and SLECs (**k**) in the blood of WT and ∆dblGATA-1 mice on d6 after *L.m*.-OVA infection. Data are mean ± SD of one representative experiment. Similar results were seen in two independent experiments with n = 5–6 mice per group. Unpaired Student’s *t* tests unless noted. NS, not significant, *p < 0.05, **p < 0.01, ***p < 0.001

The generation of memory CD8^+^ T cell pool is tightly regulated by the apoptosis or survival of precursors of memory cells. Therefore, we suspected that the impaired generation of memory CD8^+^ T cells in eosinophil-deficient mice may result from the decreased numbers of antigen-specific CD8^+^ T cells in the expansion phase (on d0–d8 p.i.) or the contraction phase (on d9–d15 p.i.). To answer this question, we first analyzed antigen-specific CD8^+^ T cells in the spleens, MLNs and blood of *L.m*.-OVA infected WT and ∆dblGATA-1 mice on d8 and d15 p.i.. Notably, ∆dblGATA-1 mice had reduced proportions and counts of antigen-specific CD8^+^ T cells in the spleens on d8 p.i. compared to WT mice (Fig. 2a). ∆dblGATA-1 mice also had decreased proportions of antigen-specific SLECs in the spleens on d8 p.i. (Fig. 2b). We detected similar changes of antigen-specific CD8^+^ T cells and SLECs in the blood as in the spleens (Fig. 2c, d). Proportions of antigen-specific CD8^+^ T cells in the MLNs between ∆dblGATA-1 and WT mice on d8 p.i. showed no difference (Supplementary Fig. 2a). Proportions and counts of CD4^+^ T cells in the spleens, MLNs and blood were not affected in ∆dblGATA-1 mice on d8 p.i. (Supplementary Fig. 2b). Likewise, the proportions and counts of splenic antigen-specific CD8^+^ T cells in ∆dblGATA-1 mice were lower than WT mice on d15 p.i. (Fig. 2e). While in the blood (Fig. 2f) and MLNs (Supplementary Fig. 2c), the proportions of antigen-specific CD8^+^ T cells showed no difference. Moreover, WT and ∆dblGATA-1 mice were transferred with CD45.1^+^ CD8^+^ OT-I T cells before being infected with *L.m*.-OVA (Supplementary Fig. 2d). ∆dblGATA-1 mice exhibited the reduced proportions and counts of CD8^+^Tet^+^OT-I T cells and SLECs in the spleens (Fig. 2g–i) and blood (Fig. 2j, k) on d6 p.i. compared to WT mice. In summary, ∆dblGATA-1 mice exhibited impaired antigen-specific CD8^+^ T cell response in all three phases after primary infection, and had defective CD8^+^ T cell recall response after secondary infection. These results suggested that eosinophils are important for generation of the antigen-specific CD8^+^ T cells during infection.

### Eosinophils deficiency enhances CD8^+^ T cell apoptosis and decreases mouse survival upon infection

**Fig. 3:**
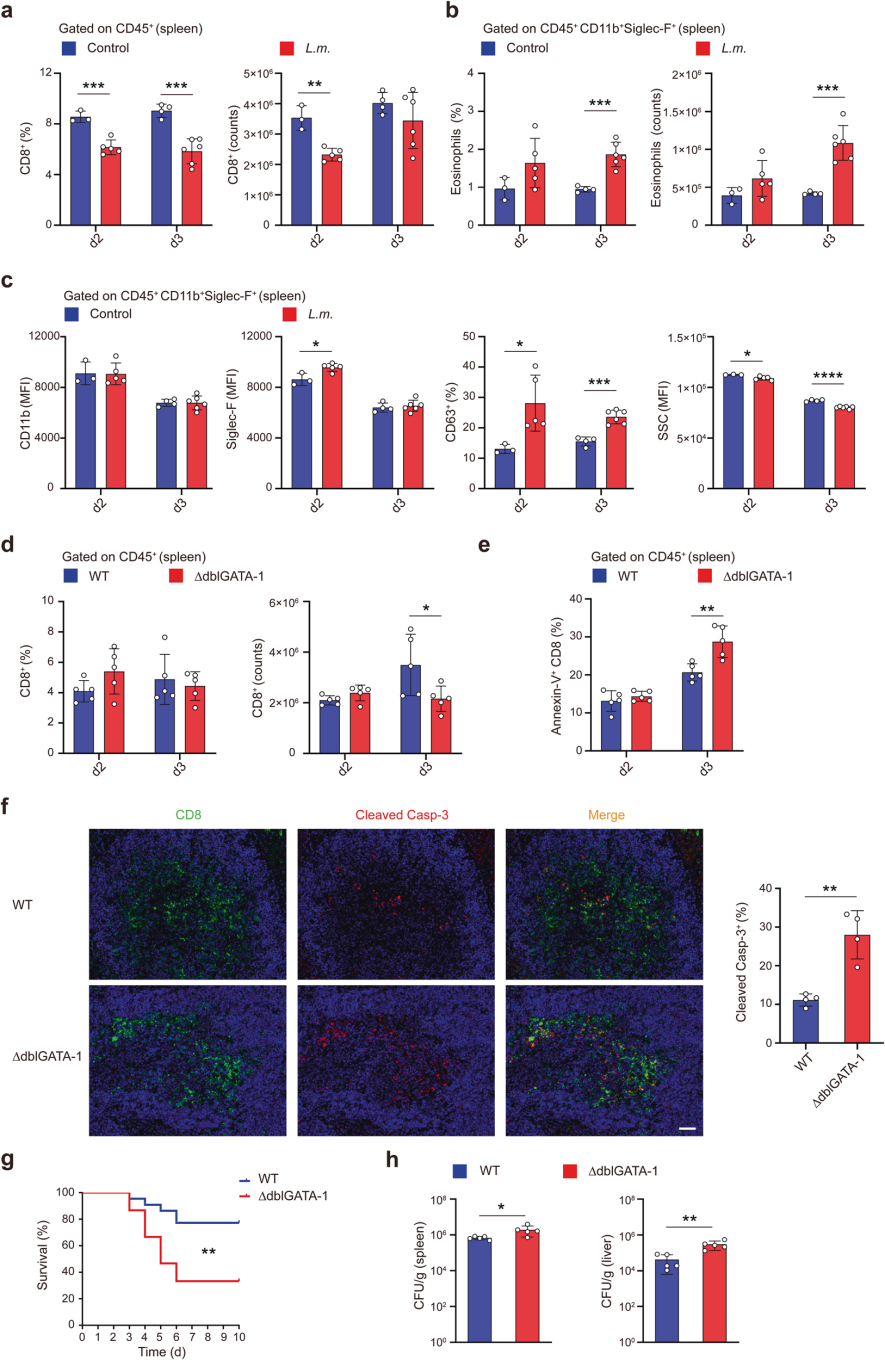
Eosinophils deficiency enhances CD8^+^ T cell apoptosis and decreases mouse survival upon infection. **a**, **b** Statistical analysis of the percentages and the counts of splenic CD8^+^ T cells (**a**) and eosinophils (**b**) in WT mice during the early stage of *L.m*. infection. **c** Activation state of splenic eosinophils assessed by CD11b and Siglec-F expression, degranulation assessed by CD63 expression and granularity change assessed by SSC. **d**, **e** Statistical analysis of the percentages and the counts (**d**) and Annexin-V expression (**e**) of splenic CD8^+^ T cells in WT and ∆dblGATA-1mice during the early infection. **f** Confocal microscopy of the cleaved caspase-3 (red) staining of splenic CD8^+^ T cells (green) in WT and ∆dblGATA-1 mice on d3 after *L.m*. infection. Scale bar, 40 μm. Right, frequency of CD8^+^ T cells localizing together with cleaved Caspase-3^+^ cells. **g** Survival curve of WT and ∆dblGATA-1 mice injected with a half-lethal dose of *L.m*.-OVA. **p < 0.01 (Log-rank (Mantel-Cox) test). **h** Bacterial load in the spleen and liver of WT and ∆dblGATA-1 mice was determined on d3 p.i.. Data are mean ± SD of one representative experiment. Similar results were seen in two independent experiments with *n* = 3–6 mice per group. Unpaired Student’s t tests unless noted. *p < 0.05, **p < 0.01, ***p < 0.001, ****p < 0.0001

*L.m*. infection usually results in rapid and extensive depletion of lymphocytes surrounding the periarteriolar lymphoid sheaths, characterized by apoptotic lesions in the spleens, lymph nodes, livers, and brains of *L.m*.-infected mice.^29^ Since apoptosis of splenic lymphocytes mostly occurred at 48 h post infection,^30^ we then analyzed the dynamic changes of CD8^+^ T cells and eosinophils during early *L.m*. infection (d0–d3). The proportions and counts of splenic and blood CD8^+^ T cells were decreased on d2 and d3 p.i. (Fig. 3a and Supplementary Fig. 3a), whereas eosinophils were increased significantly in the spleens (Fig. 3b) and decreased in the blood (Supplementary Fig. 3b). Recent studies identified a new PD-L1^+^CD80^+^ eosinophils subset termed as active eosinophils (A-Eos) which highly express Siglec-F, CD63 and SSC, and have antibacterial and regulatory functions in gastrointestinal inflammation.^31^ We observed eosinophils exhibited higher activation (CD11b and Siglec-F) and secretory activity (CD63), and less granularity (SSC) upon *L.m*. infection in the spleens or blood (Fig. 3c and Supplementary Fig. 3c). Therefore, *L.m*. infection resulted in the functional activation of eosinophils during the early stage.

So we next wondered whether eosinophils affected the apoptosis of CD8^+^ T cells during early stage of infection. We observed decreased numbers of CD8^+^ T cells in ∆dblGATA-1 mice (Fig. 3d), and higher expression of Annexin-V (Fig. 3e) and cleaved caspase-3 (Fig. 3f) on splenic CD8^+^ T cells of ∆dblGATA-1 mice. Therefore, eosinophils protect CD8^+^ T cells from apoptosis in vivo during the early stage of *L.m*. infection. Importantly, when injected with a half-lethal dose of *L.m*., ∆dblGATA-1 mice had decreased survival than the WT mice after 3 days p.i. (Fig. 3g), which was likely related with the extensive apoptosis of CD8^+^ T cells and attenuated ability to eliminate the invaded bacteria. In addition, ∆dblGATA-1 mice had reduced proportions and counts of NK cells (Supplementary Fig. 3d) and impaired secretion of IFN-γ but not granzyme B and perforin by NK cells (Supplementary Fig. 3e), and increased bacterial burdens in spleen and liver after *L.m*. infection (Fig. 3h), suggesting potential interaction between eosinophils and NK cells during infection remaining to be investigated. Our results suggest that eosinophils can prevent CD8^+^ T cells from apoptosis during early infection and promote long-term survival of infected mice.

### Eosinophil-derived cytokines inhibit CD8^+^ T cell apoptosis upon infection

**Fig. 4:**
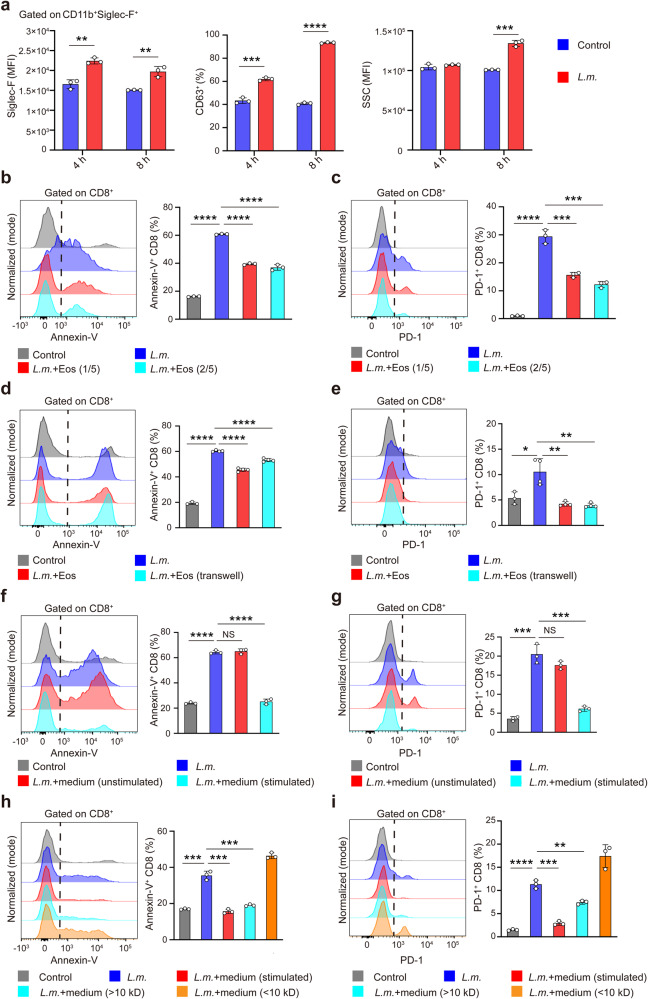
Eosinophil-derived soluble factors inhibit *L*.*m*.-induced CD8^+^ T cell apoptosis. **a** Eosinophils infected with *L.m*. for 8 h. Statistical analysis of CD63^+^ eosinophils, MFI of Siglec-F and SSC. **b**, **c** Representative FACS image and statistical analysis of Annexin-V (**b**) and PD-1 (**c**) expression in CD8^+^ T cells co-cultured with eosinophils at indicated ratio (5:1 or 5:2) after *L.m*. infection. **d**, **e** Representative FACS image and statistical analysis of Annexin-V (**d**) and PD-1 (**e**) expression in CD8^+^ T cells co-cultured with eosinophils at a ratio of 5:2 in a 1 μm transwell system after *L.m*. infection. **f, g** Representative FACS image and statistical analysis of Annexin-V (**f**) and PD-1 (**g**) expression in CD8^+^ T cells co-cultured with supernatants of *L.m.-*stimulated or resting eosinophils. **h**, **i** Representative FACS image and statistical analysis of Annexin-V (**h**) and PD-1 (**i**) expression in CD8^+^ T cells co-cultured with total or fractionated cultural supernatants of eosinophils after *L.m*. infection (molecules with MW > 10 kDa or <10 kDa). Data are mean ± SD of one representative experiment. Similar results were seen in two or three independent experiments. Unpaired Student’s t tests. NS, not significant, **p < 0.01, ***p < 0.001, ****p < 0.0001

We went further to investigate how eosinophils inhibited the apoptosis of CD8^+^ T cells. We purified eosinophils from mouse bone marrow progenitors (Supplementary Fig. 4a) and then co-cultured eosinophils with CD8^+^ T cells upon *L.m*. infection in vitro. Eosinophils infected by *L.m*. exhibited significantly higher activation (CD11b and Siglec-F) and secretory activity (CD63), elevated granularity (SSC) and apoptosis (Fig. 4a and Supplementary Fig. 4b). *L.m*. infection significantly increased proportions of apoptotic CD8^+^ T cells which expressed apoptosis marker Annexin-V and exhaustion marker PD-1 (Fig. 4b, c). Co-culturing with eosinophils effectively inhibited apoptosis of CD8^+^ T cells induced by *L.m*. infection (Fig. 4b, c). By separating eosinophils and CD8^+^ T cells in different chambers of transwell system, we found that eosinophils could protect CD8^+^ T cells from apoptosis induction by *L.m*. infection as did in co-culture system (Fig. 4d, e), suggesting that inhibition of *L.m*. infection-induced apoptosis of CD8^+^ T cells by eosinophils did not require direct cell-cell contact. Indeed, we found that the supernatant of eosinophils after *L.m*. infection suppressed apoptosis of CD8^+^ T cells induced by *L.m*. infection (Fig. 4f, g). We further separated the eosinophils-derived supernatant by size fractionation and found that only the fraction containing molecules greater than 10 kDa could efficiently inhibit apoptosis of CD8^+^ T cells while that lower than 10 kDa had no such effects (Fig. 4h, i). These results suggested that soluble molecules such as cytokines and chemokines secreted by eosinophils upon infection might be responsible for inhibiting *L.m*.-induced apoptosis of CD8^+^ T cells.

### Eosinophils secrete IL-4 to inhibit CD8^+^ T cell apoptosis upon *L.m*. infection

**Fig. 5:**
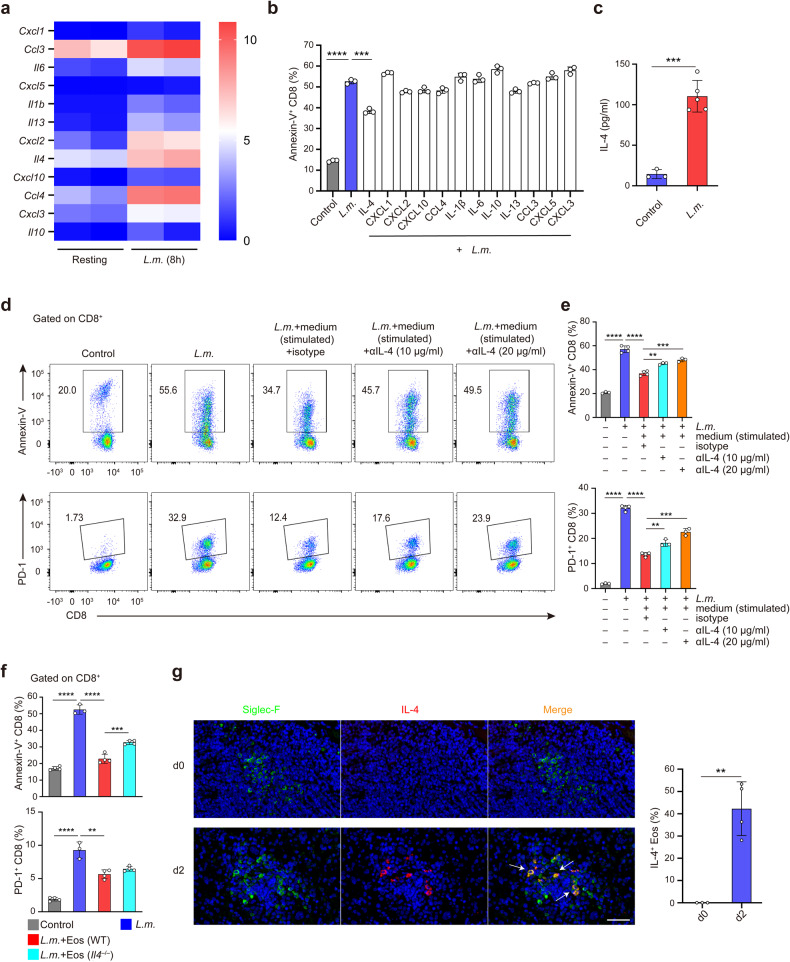
IL-4 secreted by eosinophils inhibits CD8^+^ T cells apoptosis after *L.m*. infection. **a** Heat map showing upregulated cytokine and chemokine genes in eosinophils after *L.m*. infection for 8 h in vitro (*P* adjusted <0.01 and log_2_ fold change >2), as analyzed by RNA-seq. **b** The percentages of Annexin-V^+^ CD8^+^ T cells upon *L.m.* infection for 8 h in the presence of 50 ng/ml of indicated exogenous recombinant cytokines or chemokines, measured by FACS. **c** IL-4 protein levels in the supernatant of eosinophils with or without *L.m*. infection for 8 h determined by ELISA. **d**, **e** Representative dot plots of FACS analysis of CD8^+^ T cells co-cultured with *L.m*.-stimulated eosinophils supernatant in the presence of anti-IL-4 antibody at different concentrations or isotype control after *L.m*. infection (**d**). The percentages of Annexin-V^+^ and PD-1^+^ CD8^+^ T cells were shown in (**e**). **f** Statistical analysis of Annexin-V^+^ and PD-1^+^ CD8^+^ T cells co-cultured with *Il4*^–/–^ or WT eosinophils at a 5:2 ratio after *L.m*. infection. **g** Confocal microscopy of immunofluorescence staining for IL-4 (red) and Siglec-F (green) in the spleen sections from WT mice on d0 and d2 post *L.m*. infection. Scale bar, 40 μm. Right, frequency of Siglec-F^+^ eosinophils localizing together with IL-4^+^ cells. Data are mean ± SD of one representative experiment. Similar results were seen in two or three independent experiments. Unpaired Student’s t tests. **p < 0.01, ***p < 0.001, ****p < 0.0001

We next explored the eosinophils-derived molecules responsible for inhibiting apoptosis induction of CD8^+^ T cells. We performed RNA-seq analysis of eosinophils in response to *L.m*. infection and observed increased expressions of a series of cytokine and chemokine genes in eosinophils (Fig. 5a). We selected 12 cytokines/chemokines which were increased in eosinophils upon infection and tested their effects on CD8^+^ T cell apoptosis (Fig. 5b). We found that only pretreatment of CD8^+^ T cells with exogenous recombinant IL-4 could significantly reduce proportions of Annexin-V^+^ apoptotic CD8^+^ T cells after *L.m*. infection (Fig. 5b). We further confirmed that the protein level of IL-4 increased significantly in the supernatant of eosinophils after *L.m*. infection (Fig. 5c). Moreover, addition of IL-4 neutralizing antibody into the supernatant of the *L.m*.-infected eosinophils weakened its anti-apoptotic effect on CD8^+^ T cells (Fig. 5d, e). We next co-cultured CD8^+^ T cells with IL-4-deficient (*Il4*^–/–^) or WT eosinophils upon *L.m*. infection in vitro, and found that IL-4 deficiency also weakened the anti-apoptotic effect of eosinophils on CD8^+^ T cells (Fig. 5f). However, these results demonstrated that IL-4 was partially responsible for anti-apoptotic effect, indicating other soluble granule proteins and extracellular vesicles secreted by eosinophils may participate in the process. Therefore, eosinophil-derived IL-4 could inhibit CD8^+^ T cell apoptosis induced by *L.m*. infection in vitro.

We next investigated eosinophil recruitment and IL-4 secretion in the spleen post infection in vivo. We found that the percentages and counts of splenic eosinophils reached their peak on d3–4 after *L.m*. infection and then decreased (Supplementary Fig. 5a). Thus eosinophils were recruited into the spleen at an early stage after *L.m*. infection. Moreover, immunofluorescence staining demonstrated an increased expression of IL-4 and co-localization of IL-4 with the specific maker Siglec-F of eosinophils in response to *L.m*. infection (Fig. 5g). Splenic eosinophils exhibited increased MFI of IL-4 on d2 post infection as detected by FACS (Supplementary Fig. 5b), further confirming the expression of IL-4 by eosinophils upon *L.m*. infection in vivo. Th2 cells could secrete IL-4 during microbial infection.^32^ The percentage of IL-4 expression in splenic CD4^+^ T cells on d2 post *L.m*. infection was increased (Supplementary Fig. 5c). However, there were no differences of IL-4 expression in splenic CD4^+^ T cells between ΔdblGATA-1 and WT mice on d2 post infection (Supplementary Fig. 5d). Therefore, the secretion of IL-4 by Th2 cells is not affected by eosinophils upon *L.m*. infection. Together, these results suggested that *L.m*. infection could induce IL-4 secretion by eosinophils and eosinophils-derived IL-4 mediate the inhibition of CD8^+^ T cell apoptosis during early stage of *L.m*. infection.

### IL-4 inhibits JNK/Caspase-3-mediated CD8^+^ T cell apoptosis upon *L.m*. infection

**Fig. 6:**
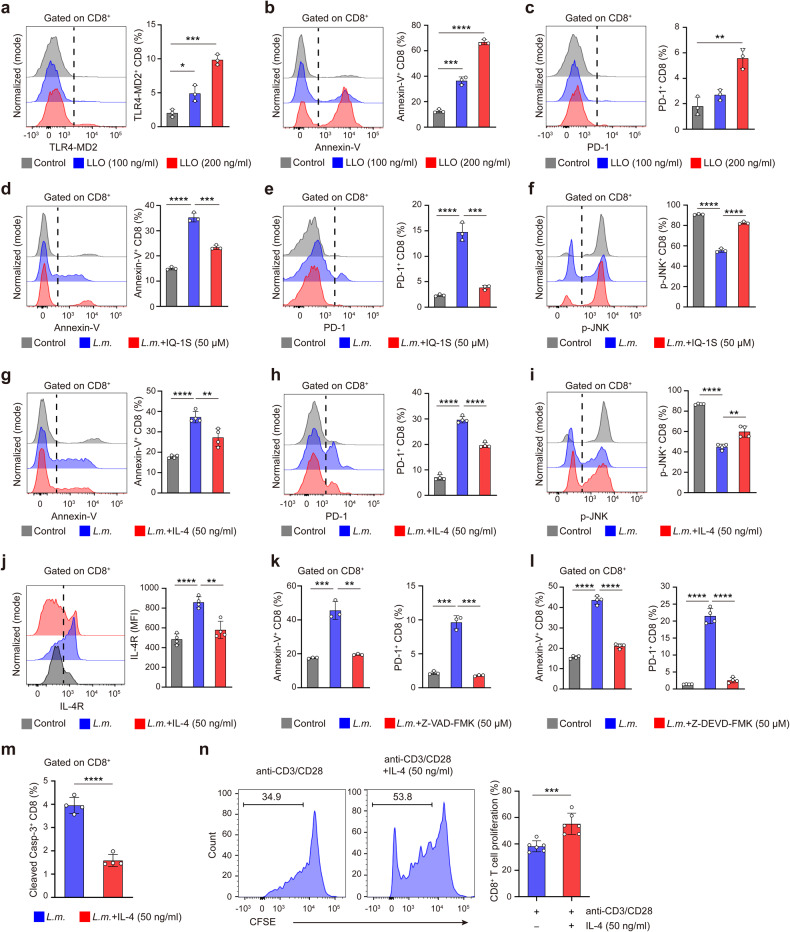
IL-4 inhibits the JNK/caspase-3-mediated CD8^+^ T cell apoptosis in response to *L.m*. infection. **a**–**c** Representative FACS image and statistical analysis of TLR4-MD2^+^ (**a**), Annexin-V^+^ (**b**) and PD-1^+^ (**c**) CD8^+^ T cells with or without LLO treatment for 4–6 h. **d**–**f** Representative FACS image and statistical analysis of Annexin-V^+^ (**d**), PD-1^+^ (**e**) and cytoplasmic p-JNK^+^ (**f**) of CD8^+^ T cells with or without JNK inhibitor IQ-1S treatment after *L.m*. infection. **g**–**i** Representative FACS image and statistical analysis of Annexin-V^+^ (**g**), PD-1^+^ (**h**) and cytoplasmic p-JNK^+^ (**i**) of CD8^+^ T cells with or without exogenous IL-4 treatment after *L.m*. infection. **j** Representative FACS image and quantification of MFI of IL-4R expression in CD8^+^ T cells with or without treatment of exogenous IL-4. **k**, **l** Proportions of Annexin-V^+^ and PD-1^+^ CD8^+^ T cells with or without Z-VAD-FMK (**k**) and Z-DEVD-FMK (**l**) treatment after *L.m*. infection as measured by flow cytometry. **m** Proportions of cleaved Caspase-3^+^CD8^+^ T cells with or without IL-4 treatment after *L.m*. infection as measured by flow cytometry. **n** CFSE analysis of proliferative CD8^+^ T cells after anti-CD3/CD28 stimulation with IL-4 treatment or not. Data are mean ± SD of one representative experiment. Similar results were seen in two or three independent experiments. Unpaired Student’s *t* tests. *p < 0.05, **p < 0.01, ***p < 0.001, ****p < 0.0001

We next explored the mechanism by which eosinophil-derived IL-4 inhibits apoptotic response of CD8^+^ T cells in responsible to *L.m*. infection. The primary virulence factor of *L.m*. is Listeriolysin O (LLO) which induces apoptosis of activated lymphocytes.^33^ Many cholesterol dependent cytolysins family members including LLO can act as TLR4 agonist to activate downstream pathway to induce apoptosis.^34^ We showed that LLO treatment activated TLR4-MD2 signaling (Fig. 6a) and induced CD8^+^ T cell apoptosis and exhaustion (Fig. 6b, c). We further employed the chemical inhibitors to block the three major TLR4 downstream pathways, JNK, P38 and NF-κB to look for the key signal pathway involved. Inhibitor vinpocetine targeting IκB kinase complex (IKK) did not affect the percentages of Annexin-V^+^CD8^+^ or PD-1^+^CD8^+^ T cells upon *L.m*. infection (Supplementary Fig. 6a, b). P38 inhibitor semapimod led to increased Annexin-V^+^ and PD-1^+^ CD8^+^ T cells upon *L.m*. infection (Supplementary Fig. 6c), indicating that P38 may promote the survival ability of CD8^+^ T cells. Notably, JNK inhibitor IQ-1S and SP600125 significantly reduced percentages of Annexin-V^+^ and PD1^+^ CD8^+^ T cells induced by *L.m*. infection (Fig. 6d, e and Supplementary Fig. 6d). *L.m*. infection decreased level of cytoplasmic p-JNK in CD8^+^ T cells, which was rescued by JNK inhibitor IQ-1S (Fig. 6f). Furthermore, exogenous treatment of IL-4 could reduce apoptosis (Fig. 6g, h), and rescue the impaired cytoplasmic p-JNK in CD8^+^ T cells (Fig. 6i). Consistently, IL-4 treatment could reduce IL-4R expression on CD8^+^ T cells upregulated by *L.m*. infection (Fig. 6j). Activation of JNK and downstream caspases was shown to induce mitochondrial-dependent apoptosis.^35^ We next addressed whether the downstream caspase pathway of JNK was influenced in CD8^+^ T cell apoptosis upon *L.m*. infection. We found that the pan-caspase inhibitor Z-VAD-FMK significantly reduced percentages of Annexin-V^+^ and PD-1^+^ CD8^+^ T cells induced by *L.m*. infection (Fig. 6k and Supplementary Fig. 7a), and the specific inhibitor of effector caspase-3 Z-DEVD-FMK treatment displayed consistent result (Fig. 6l and Supplementary Fig. 7b). In addition, IL-4 downregulated the expression of cleaved caspase-3 in CD8^+^ T cells upon infection (Fig. 6m and Supplementary Fig. 7c). Thus, IL-4 inhibits the proapoptotic JNK/caspase-3 signaling of CD8^+^ T cells in response to *L.m*. infection. Additionally, IL-4 has been shown to promote survival of B cell and proliferation of multiple immune and non-immune cells.^36,37^ We also found that IL-4 significantly increased the proliferation of CD8^+^ T cells in vitro (Fig. 6n). Together, our findings demonstrated that eosinophils produce IL-4 to inhibit JNK/Caspase-3 dependent apoptosis of CD8^+^ T cells induced by infection.

### Eosinophils secrete IL-4 to promote memory CD8^+^ T cell generation in vivo

**Fig. 7:**
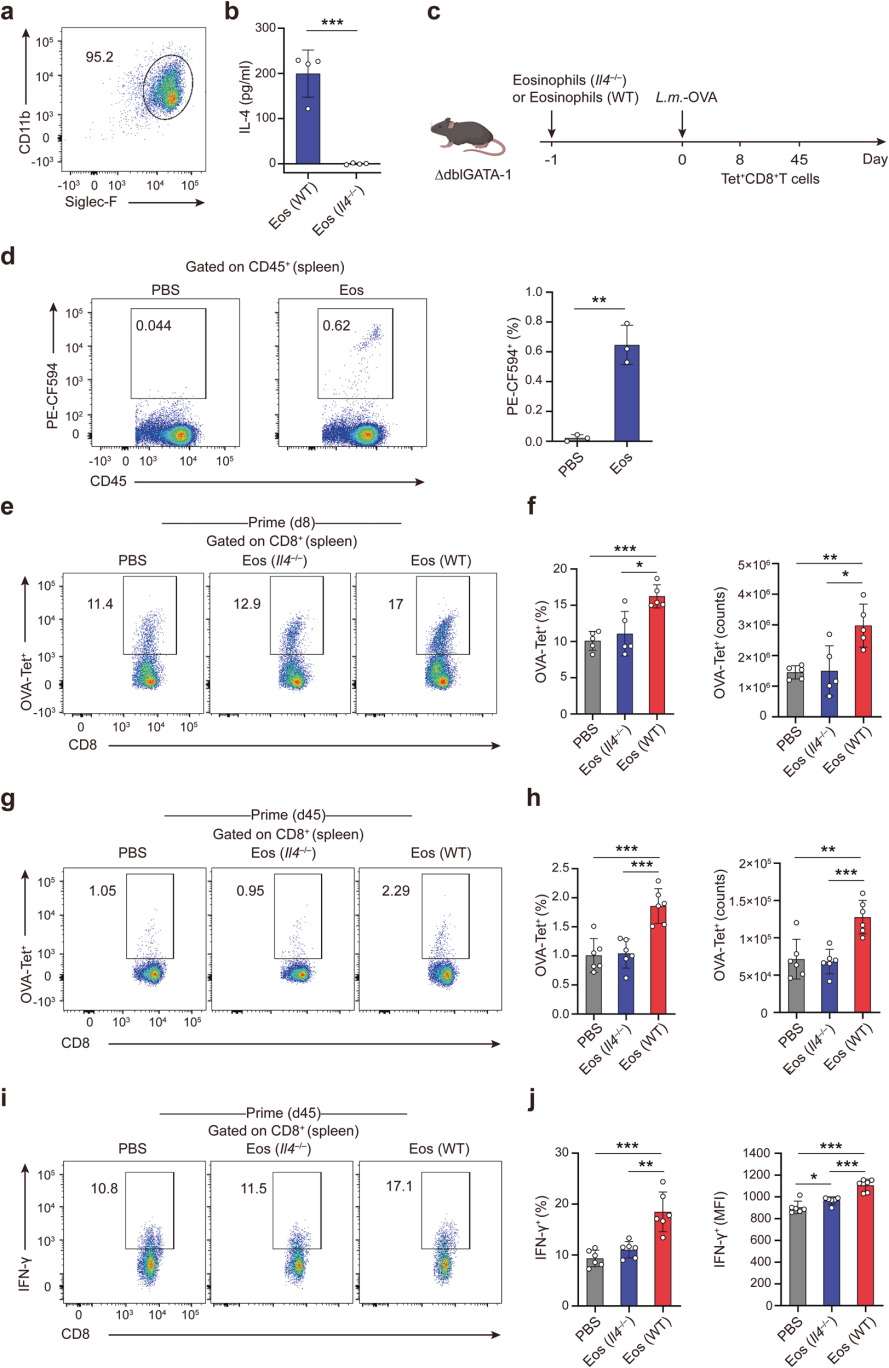
Eosinophil-derived IL-4 promotes generation of memory CD8^+^ T cell. **a** Cells differentiated from *Il4*^–/–^ mice bone marrow progenitors were harvested on day 14 and stained for CD11b and Siglec-F for FACS analysis. **b** Protein levels of IL-4 secreted by WT and *Il4*^–/–^ eosinophils after *L.m*. infection in vitro. **c** Experimental setup. ∆dblGATA-1 mice were intravenously transferred with eosinophils from WT or *Il4*^–/–^ mice, or PBS as control, at one day before *L.m*.-OVA infection, followed by OVA-Tet^+^CD8^+^ T cells analysis at d8 and d45 p.i.. **d** Mature eosinophils developed from DsRed mice bone marrow progenitors were transferred into ∆dblGATA-1 mice. Two days after injection, representative dot plots of FACS and statistical analysis of PE-CF594^+^ eosinophils in the spleens. **e**, **f** Splenocytes were collected on d8 from mice treated as in (**c**) and analyzed for OVA-Tet^+^CD8^+^ T cells by FACS (**e**). Statistical analysis of the percentages and the counts (**f**) of OVA-Tet^+^CD8^+^ T cells were shown. **g**, **h** Splenocytes were collected on d45 from mice treated as in (**c**) and analyzed for OVA-Tet^+^CD8^+^ T cells by FACS (**g**). Statistical analysis of the percentages and the counts (**h**) of OVA-Tet^+^CD8^+^ T cells were shown. **i, j** Splenocytes were collected on d45 from mice treated as in (**c**) and analyzed for IFN-γ^+^CD8^+^ T cells by FACS (**i**). Statistical analysis of the percentages of IFN-γ^+^CD8^+^ T cells and the IFN-γ MFI (**j**) were shown. Data are mean ± SD of one representative experiment. Similar results were seen in two independent experiments with *n* = 5–6 mice per group. Unpaired Student’s t tests. *p < 0.05, **p < 0.01, ***p < 0.001

Above data suggested that eosinophils deficiency impaired generation of memory CD8^+^ T cells, and that eosinophils-derived IL-4 inhibited CD8^+^ T cell apoptosis during early *L.m*. infection. We next addressed whether IL-4 derived from eosinophils during early infection was responsible for maintaining the quantity of memory CD8^+^ T cells. By using *Il4*^–/–^ mice, we found that *Il4* deficiency did not influence the development and maturation of eosinophils from bone marrow progenitor cells (Fig. 7a) and confirmed the absence of IL-4 expression or secretion in *Il4*^–/–^ eosinophils upon *L.m*. infection (Fig. 7b). We adoptively transferred *Il4*^–/–^ or WT eosinophils into ∆dblGATA-1 mice one day prior to *L.m*.-OVA infection and then analyzed splenic antigen-specific Tet^+^CD8^+^ T cells on d8 and d45 p.i. (Fig. 7c). Two days after the adoptive transfer of eosinophils into ∆dblGATA-1 mice, a significant increase of eosinophils was detected in the spleens (Fig. 7d). ΔdblGATA-1 mice transferred with WT eosinophils exhibited increased percentages and counts of OVA-specific CD8^+^ T cells compared to those injected with PBS as control on d8 and d45 p.i. (Fig. 7e–h), which confirmed that eosinophils were required for the generation of memory CD8^+^ T cells. Notably, ∆dblGATA-1 mice transferred with *Il4*^–/–^ eosinophils showed decreased proportions and counts of splenic antigen-specific CD8^+^ T cells as compared to those transferred with WT eosinophils both on d8 and d45 p.i. (Fig. 7e–h). Consistently, ∆dblGATA-1 mice transferred with *Il4*^–/–^ eosinophils displayed reduced expression of IFN-γ in CD8^+^ T cells on d45 p.i. (Fig. 7i, j). Therefore, we demonstrate that IL-4 derived from eosinophils during early stage of infection could consequently promote generation and function of memory CD8^+^ T cell.

## Discussion

The cellular and molecular mechanisms for the long-term survival of antigen-specific memory T cells remain incompletely elucidated. In this research, we showed that eosinophils play crucial roles in facilitating the formation of memory CD8^+^ T cell during bacterial infection via IL-4-mediated inhibition of apoptosis. Our study uncovers additional mechanisms for the formation of memory CD8^+^ T cells and may facilitate the development of more effective vaccines as well as T cell-based immunotherapies against infections and cancers.

A balance between anti-apoptotic and pro-apoptotic factor contributes to fine-tuned control of T cell survival and ultimately the generation of immune memory.^38^ Pro-apoptotic factor Bim regulates the apoptosis of activated T cells, therefore dampening T cell memory and pathogen clearance.^39,40^ JNK plays flexible roles in either apoptotic induction or survival maintenance, in a manner dependent on stimuli strength and quality.^41^ JNK dependent apoptosis has been proposed to be associated with activation of Bcl-2 family members, mitochondrial pathway and caspases.^35,42^ We showed that *L.m*. infection induces eosinophils to secrete IL-4 to inhibit the JNK/caspase-3-dependent apoptosis of CD8^+^ T cells at early stage, which consequently promotes generation of immune memory and protective immunity against infection. Therefore, we identified new mechanisms that promote CD8^+^ T memory via preventing cell death during early stage of activation. The downstream signaling that mediate JNK/Caspase-3-dependent apoptosis of CD8^+^ T cells regulated by eosinophil-derived IL-4 remained to be further identified.

Cytokines play essential roles in controlling the survival and memory of CD8^+^ T cells, such as IL-2,^43^ IL-7,^44^ IL-15,^45^ and IL-4.^46,47^ IL-4 derived from phagocytes was shown to promote the priming of vaccine-specific CD8^+^ T cells.^48^ A crosstalk between IL-4-producing iNKT cells and CD8^+^ T cells in the WP is important for generating short-lived effector cells upon vaccination.^49^ IL-4 also promotes the development of virtual memory CD8^+^ T (TVM) cells.^50–52^ Our results highlight that IL-4 secreted by eosinophils reduces the apoptosis of CD8^+^ T cells in early stage of infection and subsequently promotes CD8^+^ T cell memory. Thus, our study uncovers the ways of IL-4-dependent cell-cell communication underlying early stage of infection to promote long-term memory, which could be exploited for development of new vaccines with more potency for infectious diseases.

Eosinophils exhibit diversified function in regulating T cell responses and are implicated in a wide range of pathologies. Eosinophils extracellular traps were shown to promote Th2 immune response and correlate with the severity of asthma.^15,53^ It was recently reported that intratumoral eosinophils could activate CD8^+^ T cells and thus upregulates the effects of immune checkpoint blockade.^54^ To our knowledge, we provide the first evidence that splenic eosinophils and IL-4 functionally promote the CD8^+^ T cell memory response in bacterial infection.

### Limitations of the study

Our findings suggest eosinophils could promote the generation of CD8^+^ T cell memory to potentiate anti-bacterial immunity. It has been shown that eosinophils can promote the maintenance of bone marrow long-lived plasma cells.^55^ Therefore, we propose that eosinophils could be reinforced or activated for enhanced vaccine efficiency against infection. Further investigations will be required to determine the strategies and effects of combining eosinophils or eosinophil-activating adjuvants to optimize vaccine potency. Moreover, it should also be noted that eosinophils are potent in releasing cationic proteins and cytotoxic cytokines, causing tissue pathogenesis of inflammatory diseases. Therefore, the effects of eosinophil-based adjuvant in modulating multiple aspects of tissue homeostasis and inflammatory response remain to be further investigated.

## Materials and Methods

### Mice

C57BL/6 mice were obtained from Joint Ventures Sipper BK Experimental Animal (Shanghai, China). DsRed (JAX:006051), OT-I (JAX:003831), B6 CD45.1 (JAX:002014) and ∆dblGATA-1 (JAX:005653, BALB/c background) mice were obtained from Jackson Laboratories. ∆dblGATA-1 mutant mice on the C57BL/6 background were obtained through eight successive generations of backcrossing with C57BL/6 mice. CD45.1 OT-I mice were obtained by crossing CD45.1 mice and OT-I mice. *Il4*^–/–^ mice (NM-KO-190503, C57BL/6 background) were obtained from the Shanghai Model Organisms Centre (Shanghai, China). The mice were maintained under specific pathogen-free conditions and selected to ensure age and sex matching in experiments. All mouse experiments were approved by the Scientific Investigation Board of Naval Medical University.

### Antibodies and reagents

Cell Activation Cocktail (with Brefeldin A), Fixation Buffer, APC-Cy7 anti-mouse CD45 (30-F11), PE-Cy7 anti-mouse CD45.1 (A20), PE-Cy7/ Percp-cy5.5 anti-mouse CD4 (RM4-5), APC anti-mouse IFN-γ (XMG1.2), APC anti-mouse PD-1 (29F.1A12), APC/BV605 anti-mouse KLRG1 (2F1), APC anti-mouse CX3CR1 (SA011F11), APC/AF488 anti-mouse CD11b (M1/70), Percp-cy5.5/BV421 anti-mouse CD8a (53-6.7), BV421 anti-mouse Siglec-F (S17007L), purified anti-mouse IL-4 antibody (11B11), PE-Cy7 anti-mouse TLR4-MD2 (MTS510), PE anti-mouse IL-4 (11B11), rmCXCL3, rmCXCL5 were purchased from Biolegend. H-2Kb OVA-(SIINFEKL)-Tetramer-PE, FITC anti-mouse CD8 (KT15) were purchased from MBL. Cytokines rmIL-4, rmCXCL1, rmSCF, rmCXCL2, rmIL-5, rmCXCL10, rmCCL4, rmIL-1β, rmIL-6, rmFLT3L, rmIL-10, rmIL-13, rmCCL3 were purchased from Peprotech. BV421 anti-mouse CD127 (A7R34), PE-CF594 anti-mouse CD3e (145-2C11), Purified rat anti-mouse Siglec-F (E50-2440), Perm/Wash Buffer, PE/FITC AnnexinV Apoptosis were purchased from BD Biosciences. PE-Cy7 anti-mouse Granzyme B (NGZB), FITC anti-mouse CD3e (145-2C11), APC anti-mouse KLRG1 (2F1), PE-eFluor610 anti-mouse NK1.1 (PK136), APC anti-mouse PD-1 (J34), PE anti-mouse Perforin (eBioOMAK-D) and Brefeldin A were purchased from eBioscience. Recombinant Listeriolysin-O was purchased from ProSpec-Tany. IQ-1S free acid, SP600125, Semapimod, Vinpocetine, Z-VAD-FMK and Z-DEVD-FMK were purchased from MCE. Phospho-SAPK/JNK (Thr183/Tyr185, 81E11) Rabbit mAb, cleaved caspase-3 (Asp175, D3E9) Rabbit mAb and anti-rabbit IgG (AF488) were purchased from CST.

### Cell cultures and induction of apoptosis

CD8^+^ T cells were isolated from spleen by using a mouse CD8^+^ T cells Enrichment kit (STEMCELL Technologies). 1 × 10^5^ CD8^+^ T cells were cultured in RPMI 1640 medium (10% FBS). 1 × 10^6^ CFU *L.m*.-OVA were added for inducing apoptosis.

### Eosinophil cultures and cell transfer

Eosinophils were differentiated from mouse bone marrow progenitors as shown in the previous study.^56^ Briefly, bone marrow cell suspensions were cultured in DMEM/F12 (Gibco) with 20% FBS, 100 ng/ml rmSCF and 100 ng/ml rmFLT3L from d0 to d4. Cultures were replaced on d4 with medium containing 10 ng/ml rmIL-5 until d14. Then we collected cells and tested the purity by flow cytometry. WT or *Il4*^–/–^ eosinophils were harvested and 1 × 10^7^ cells were injected i.v. into ∆dblGATA-1 mice.

### In vitro co-culture and transwell experiments

For co-culture system, 1 × 10^5^ CD8^+^ T cells were cultured with eosinophils at 5:1 or 5:2 ratio in 96-well U-bottomed plates. For transwell system, CD8^+^ T cells were separated from eosinophils at a 5:2 ratio by 1 μm transwell (Corning 96-Well HTS-Transwell plates), cultured with the same concentration of *L.m*.-OVA. The supernatant of eosinophils was collected from 1 × 10^6^ cells with *L.m*.-OVA infection for 8 h and separated into two fractions using ultrafiltration device Amicon Ultra (3 kDa molecular weight cutoff, Millipore).

### Protein analysis

1 × 10^6^ eosinophils were seeded in 1 ml medium and stimulated by *L.m*.-OVA for 8 h. IL-4 secretion were measured by ELISA assay according to the instruction protocol (R&D Systems).

### Flow cytometry

Cells were stained with anti-AnnexinV, CD8, PD-1, CD11b, Siglec-F, TLR4-MD2, H-2Kb OVA-(SIINFEKL)-Tetramer, KLGR1, CX3CR1, CD127, CD45, CD4, CD3, NK1.1, respectively. For intracellular staining, cells were stimulated with Cell Activation Cocktail (IFN-γ) or Brefeldin A (IL-4) for 4–6 h at 37 °C in a 5% CO_2_ incubator. Samples were firstly fixed with intracellular fixation buffer for 30 min at 4 °C in the dark. Anti-IFN-γ, anti-IL-4, anti-Granzyme B, anti-Perforin, anti-Phospho-SAPK/JNK and anti-cleaved caspase-3 were diluted in permeabilization buffer and incubated for 1 h. If necessary, cells were stained with AF488 anti-rabbit IgG antibody for 1 h. Cells were analyzed on a LSRII Fortessa (BD Biosciences). Flow-cytometric analyses were performed using FlowJo software (Tree Star).

### Cell proliferation assay

1 × 10^5^ splenic CD8^+^ T cells labeled with 0.4 μM 5(6)-CFDAN-succinmidyl ester (CFSE) (MCE) were co-cultured with 1 × 10^5^ anti-CD3/CD28 beads (Invitrogen, USA) with IL-4 treatment or not for 96 h. Proliferation was monitored by flow cytometry on a LSRII Fortessa.

### RNA sequencing

Total RNA was obtained using TRIzol, and sequenced by Annoroad Gene Technology Co.Ltd. (Beijing, China). Sequencing libraries were generated using NEBNext Ultra RNA Library Prep Kit for Illumina (#E7530L, NEB, USA). Index codes were added to attribute sequences to each sample.

### Determination of bacterial load (CFU) in organs

Tissues were homogenized and lysed with 0.1% Triton X-100, and 10-fold serial dilutions were plated on BHI agar plates. The plates were incubated at 37 °C for 24–36 h, and the CFU per gram were enumerated.

### Confocal microscopy

Paraffin-embedded spleen sections were blocked with either 10% donkey serum or 3% BSA for 30 min, incubated with mouse anti-Siglec-F, anti-CD8, anti-cleaved caspase-3 and anti-IL-4 antibodies overnight at 4 °C, followed by incubating with secondary antibodies at room temperature (RT) for 50 min. Following another round of washing, the sections were incubated with DAPI at RT for 10 min. Finally, the sections were subjected to image acquisition using a Leica TCS SP8 confocal laser microscope. Image processing was conducted using Leica Application Suite X software.

### Infections and OT-I cells transfer

Virulent *L. monocytogenes* expressing OVA protein (*L.m*.-OVA) was kindly provided by Dr. Hao Shen (University of Pennsylvania School of Medicine, Philadelphia, PA).^57,58^ A total of 3 × 10^6^ CFU *L.m*.-OVA for primary and 6 × 10^6^ CFU for half-lethal dose were diluted in 100 μl PBS and injected intraperitoneally (i.p.) into ∆dblGATA-1 mice and WT littermates. For secondary infection, 1.5 × 10^7^ CFU were diluted in 100 μl PBS and injected i.p.

For adoptive transfer, 1 × 10^5^ CD45.1^+^CD8^+^ OT-I T cells were transferred intravenously (i.v.) to WT and ∆dblGATA-1 mice. One day later, the mice were injected i.p. with 3 × 10^6^ CFU *L.m*.-OVA.

### Statistics analysis

Statistical analyses were performed with unpaired Student’s *t*-test. Log-rank test was used to estimate the statistical significance of Kaplan–Meier survival curves. *P* value < 0.05 was considered significant.

### Supplementary information

SUPPLEMENTAL MATERIAL

## Data Availability

RNA-seq data have been deposited in the NCBI’s Gene Expression Omnibus (GEO) with the accession no. GSE220480. All data supporting the findings of this study are available from the corresponding author on reasonable request.
