# Supplementary material for: Eosinophils promote CD8+ T cell memory generation to potentiate anti-bacterial immunity
Source: Signal Transduct Target Ther. 2024 Feb 28;9:43. doi: 10.1038/s41392-024-01752-0 (PMC10899176; doi:10.1038/s41392-024-01752-0)
Supplement: Supplementary file 1 — SUPPLEMENTAL MATERIAL [file 41392_2024_1752_MOESM1_ESM.docx]

Supplementary Materials for

**Eosinophils promote CD8^+^ T cell memory generation to potentiate anti-bacterial immunity**

Jun Zhou^1,2^, Jiaqi Liu^3^, Bingjing Wang^3^, Nan Li^2^, Juan Liu^2^, Yanmei Han^2,*^ and Xuetao Cao^1,2,3,4,*^

Correspondence to:

Yanmei Han (hanyanmei@immunol.org) or Xuetao Cao (caoxt@immunol.org)

**This PDF file includes:**

Figures. S1 to S7


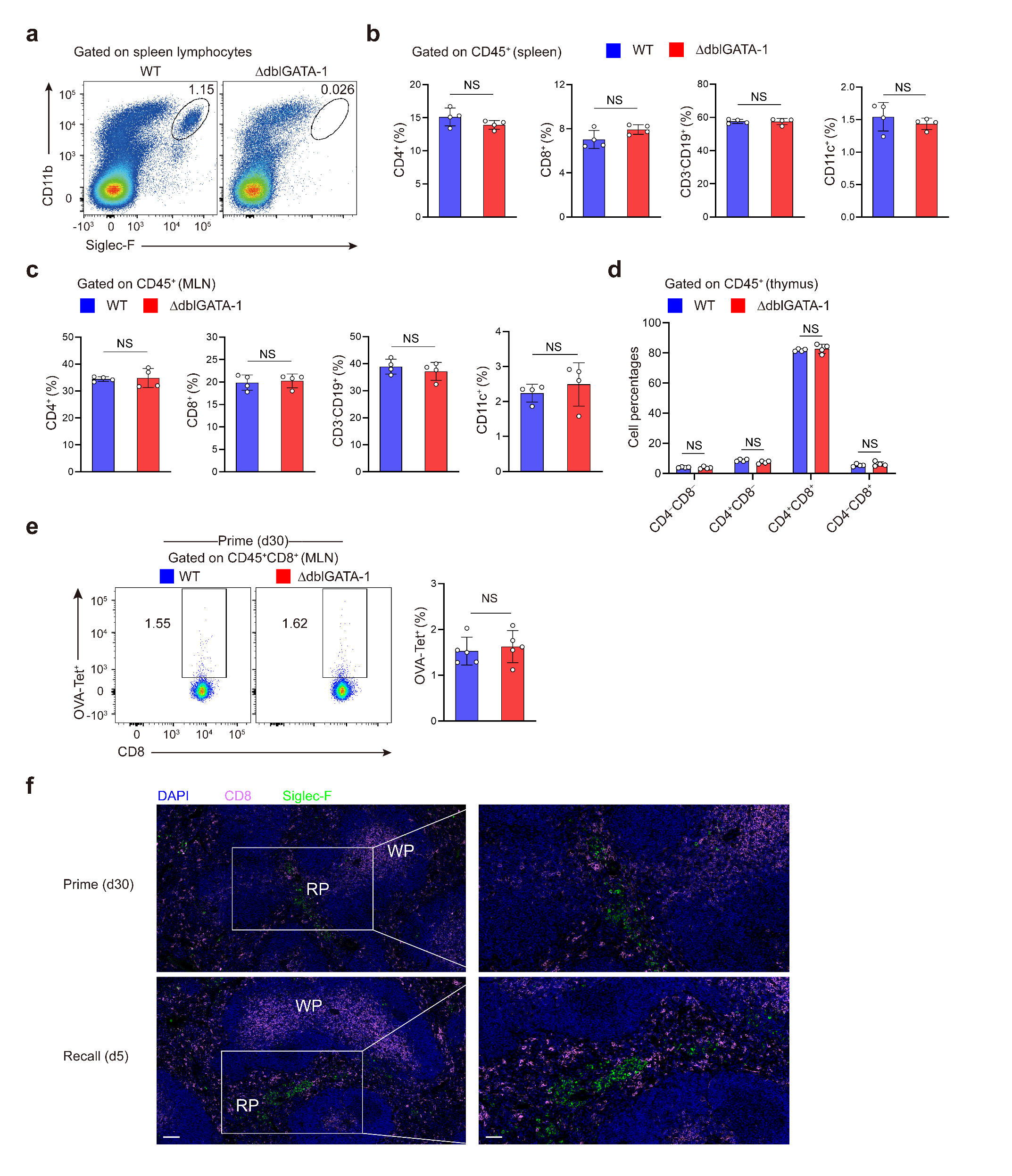


Figure. S1.

**The development of T cells is intact in ∆dblGATA-1 mice. a** FACS analysis of splenic eosinophils stained for CD11b and Siglec-F in WT and ∆dblGATA-1 mice. **b, c** Statistical analysis of CD4^+^ T cells, CD8^+^ T cells, CD3^-^CD19^+^ B cells and CD11c^+^ DCs in the spleen (**b**) and MLNs (**c**) of WT and ∆dblGATA-1 mice. **d** Statistical analysis of thymic T cells state of differentiation of WT and ∆dblGATA-1 mice. **e** Representative dot plots and statistical analysis of OVA-Tet^+^CD8^+^ T cells in the MLNs of WT and ∆dblGATA-1 mice on d30 after *L.m.*-OVA infection. **f** Confocal microscopy of splenic Siglec-F^+^ eosinophils (green) and CD8^+^ T cells (pink) in WT mice on d30 after primary infection and on d5 after secondary infection. Scale bar, 100 μm left, 40 μm right. Data are mean ± SD of one representative experiment. Similar results were seen in two independent experiments with n = 4–6 mice per group. Unpaired Student’s t tests unless noted. NS, not significant.


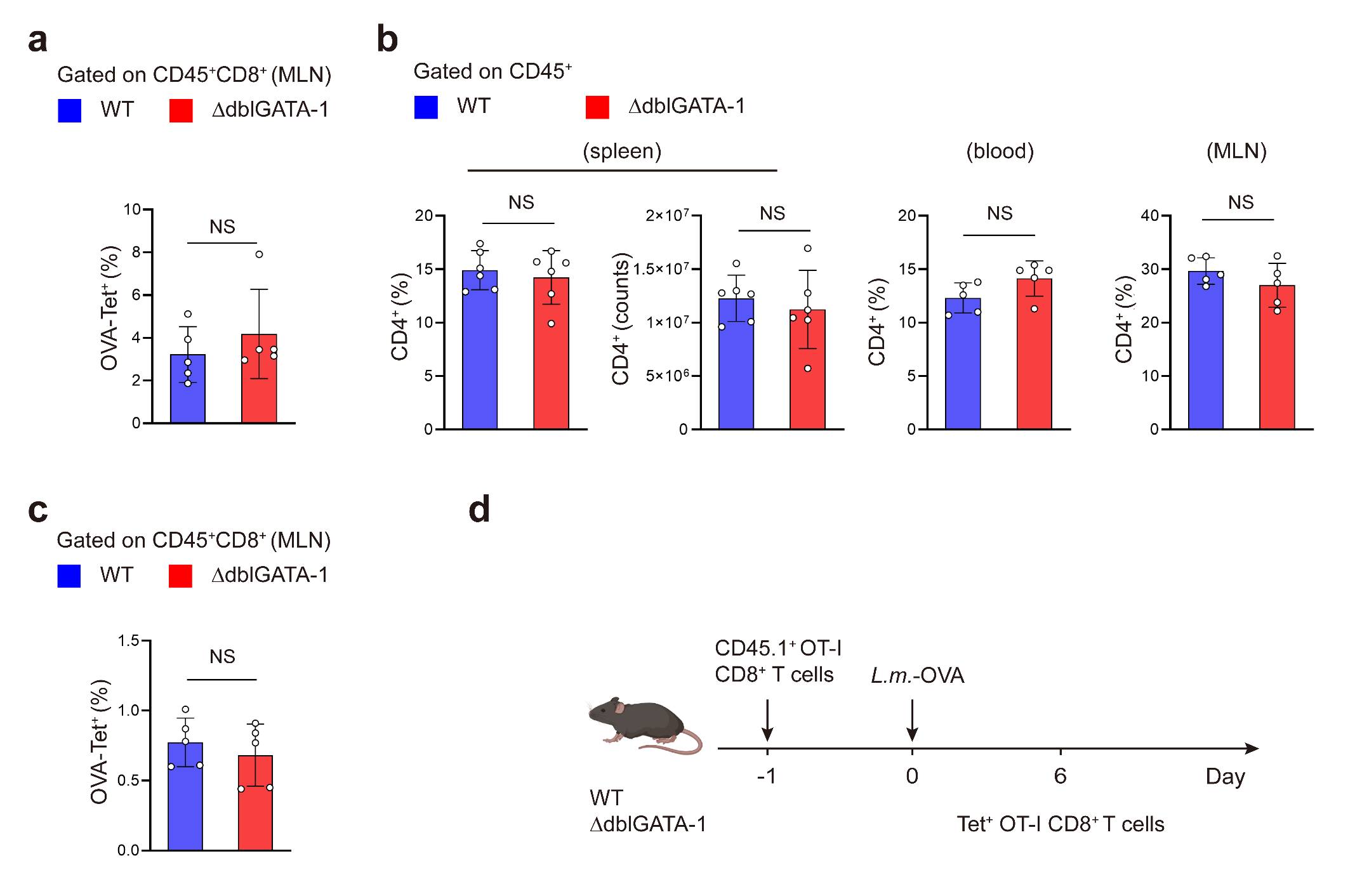


Figure. S2.

**CD4^+^ T cell responses are not affected in ∆dblGATA-1 mice after *L.m.* infection. a** Statistical analysis of OVA-Tet^+^CD8^+^ T cells in the MLNs of WT and ∆dblGATA-1 mice on d8 after *L.m.*-OVA infection. **b** Statistical analysis of the percentages and the counts of CD4^+^ T cells in the spleens, blood and MLNs of WT and ∆dblGATA-1 mice at d8 p.i.. **c** Statistical analysis of OVA-Tet^+^CD8^+^ T cells in the MLNs of WT and ∆dblGATA-1 mice on d15 after *L.m.*-OVA infection. **d** Experimental setup. WT and ∆dblGATA-1 mice were intravenously transferred with 1×10^5^ CD45.1^+^ CD8^+^ OT-I T cells one day prior to *L.m*.-OVA infection, followed by CD45.1^+^OVA-Tet^+^CD8^+^ T cells analysis at d6. Data are mean ± SD of one representative experiment. Similar results were seen in two independent experiments with n = 5–6 mice per group. Unpaired Student’s t tests unless noted. NS, not significant.


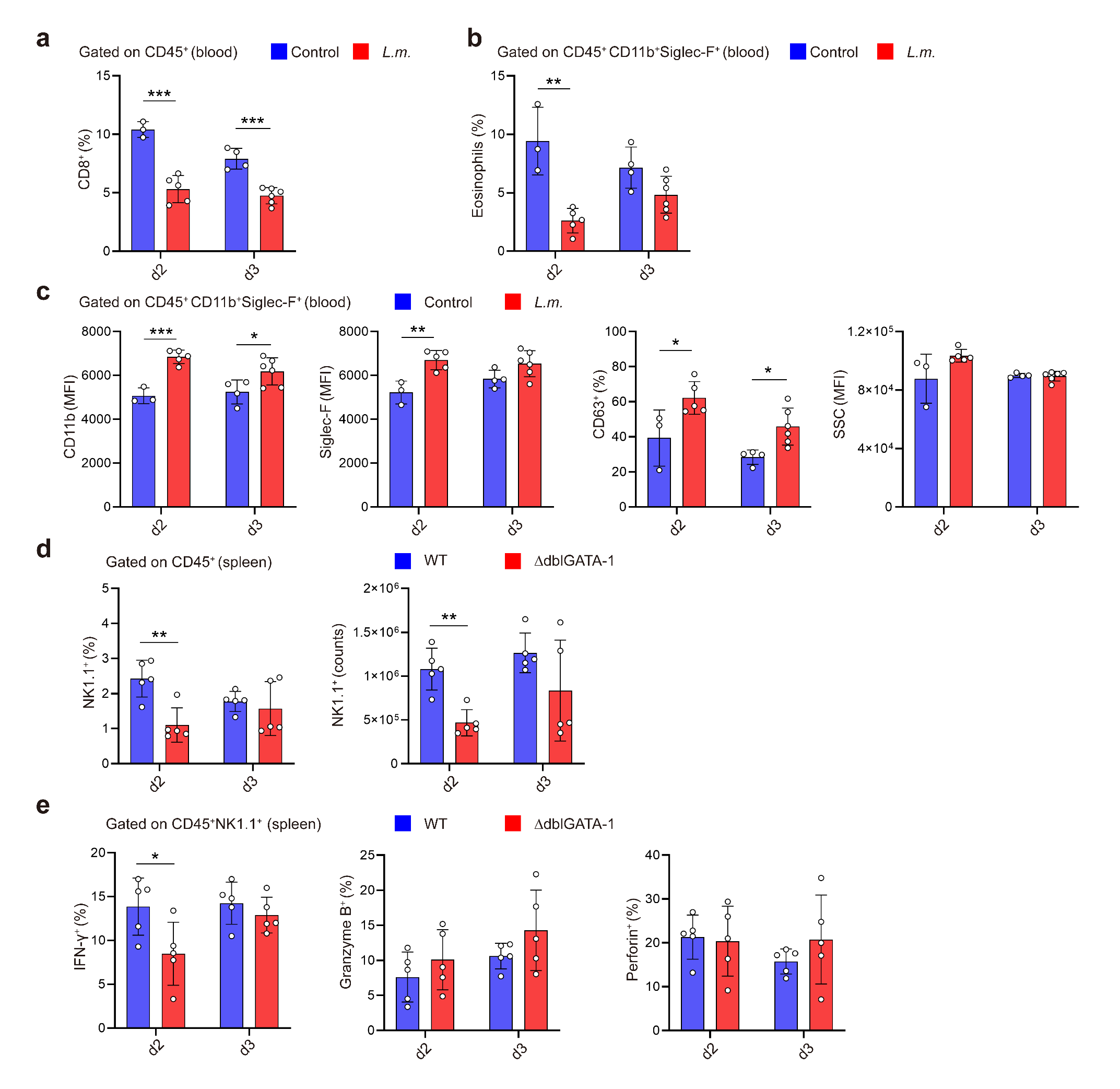


Figure. S3.

**Eosinophils deficiency enhances CD8^+^ T cell apoptosis and decreases mouse survival upon infection. a**, **b** Statistical analysis of the percentages of peripheral blood CD8^+^ T cells (**a**) and eosinophils (**b**) in WT mice during the early stage of *L.m.* infection. **c** Activation state of blood eosinophils assessed by CD11b and siglec-F expression, frequencies of degranulated eosinophils identified by CD63 expression and granularity assessed by SSC. **d** Statistical analysis of the percentages and the counts of splenic NK1.1^+^ T cells in WT mice and ∆dblGATA-1mice during the early infection. **e** Statistical analysis of and IFN-γ, granzyme B and perforin expression of splenic NK1.1^+^ T cells in WT mice and ∆dblGATA-1mice during the early infection. Data are mean ± SD of one representative experiment. Similar results were seen in two independent experiments with n = 3–6 mice per group. Unpaired Student’s t tests unless noted. NS, not significant, *p < 0.05, **p < 0.01, ***p < 0.001.


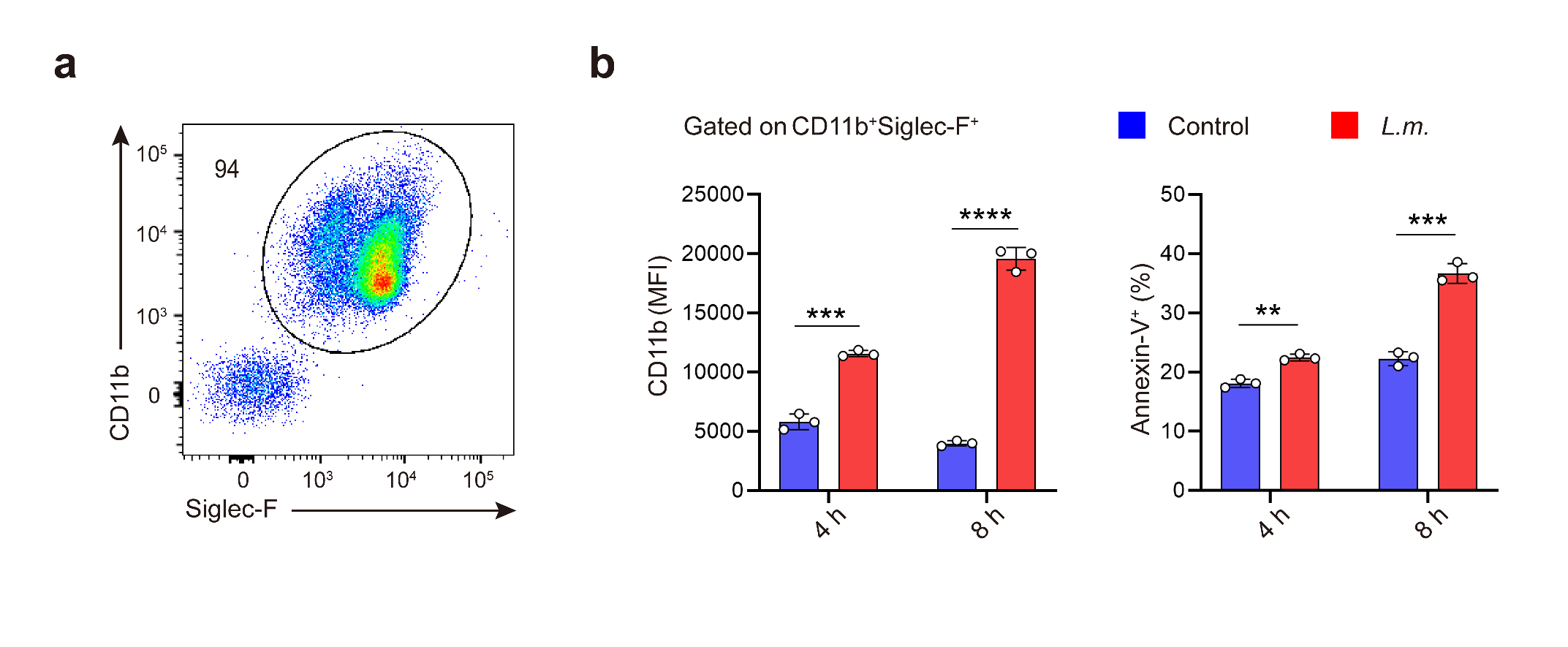


Figure. S4.

**Eosinophils exhibits higher activation and apoptosis upon *L.m* infection *in vitro*. a** Cells differentiated from mouse bone marrow progenitors were harvested on day 13 and stained for CD11b and Siglec-F for FACS analysis. **b** Eosinophils infected with *L.m.* for 8 hours. Statistical analysis of MFI of CD11b and Annexin-V^+^ eosinophils. Similar results were seen in two or three independent experiments. Unpaired Student’s t tests. **p < 0.01, ***p < 0.001, ****p < 0.0001.


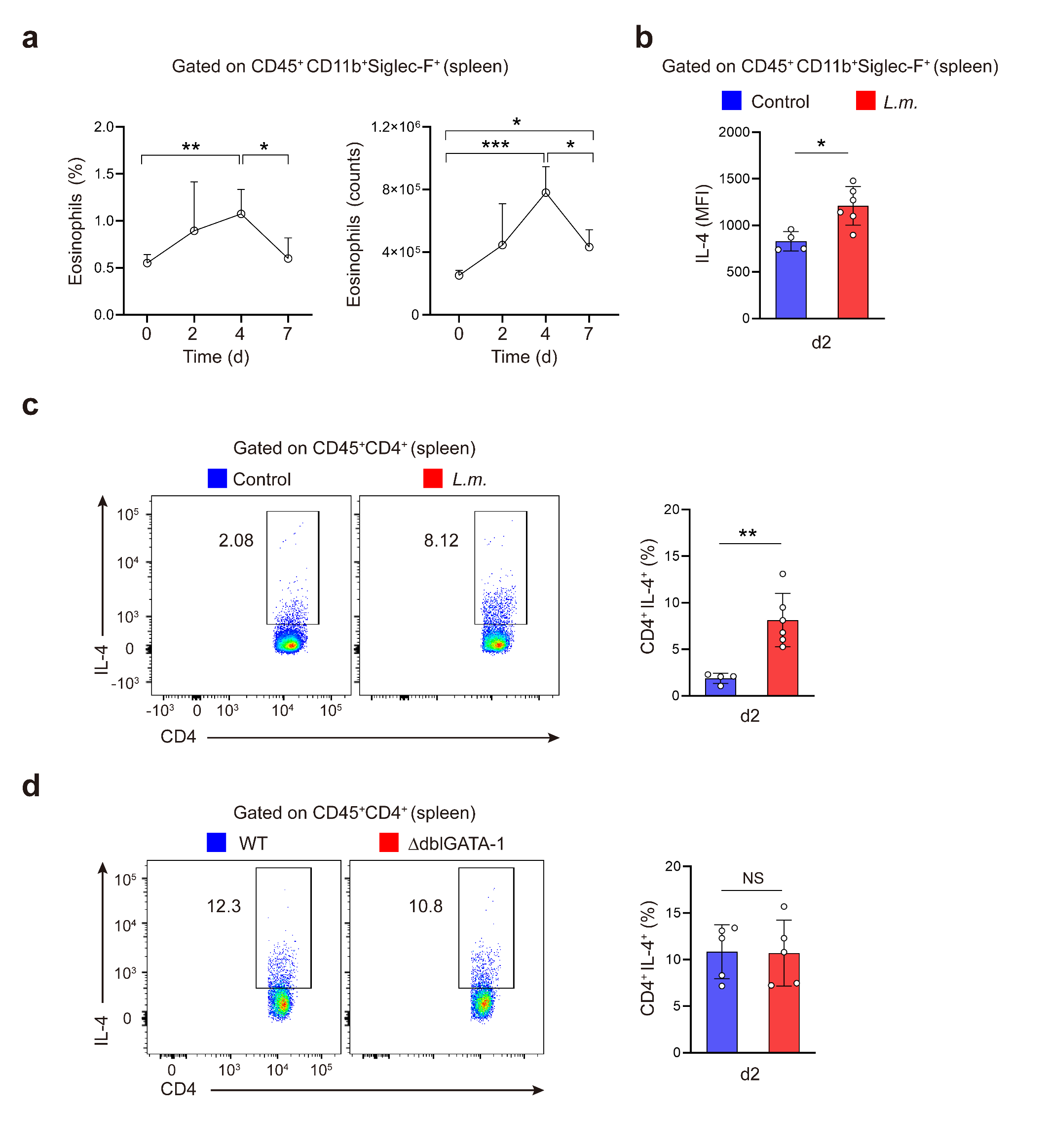


Figure. S5.

**Eosinophils secrete IL-4 to inhibit CD8^+^ T cell apoptosis upon *L.m.* infection. a** Kinetic analysis of the percentages and the counts of splenic eosinophils in WT mice after *L.m.*-OVA infection. **b** Statistical analysis of IL-4 MFI of splenic CD11b^+^Siglec-F^+^ eosinophils on d2 after *L.m.*-OVA infection. **c** Representative dot plots and statistical analysis of IL-4^+^CD4^+^ T cells in the spleen of WT mice on d0 and d2 after *L.m.*-OVA infection. **c** Representative dot plots and statistical analysis of IL-4^+^CD4^+^ T cells in the spleen of WT and ∆dblGATA-1 mice on d2 after *L.m.*-OVA infection. **d** Representative dot plots and statistical analysis of IL-4^+^CD4^+^ T cells in the spleen of WT and ∆dblGATA-1 mice on d2 after *L.m.*-OVA infection. Similar results were seen in two independent experiments with n = 4–6 mice per group. Unpaired Student’s t tests unless noted. NS, not significant, *p < 0.05, **p < 0.01, ***p < 0.001.


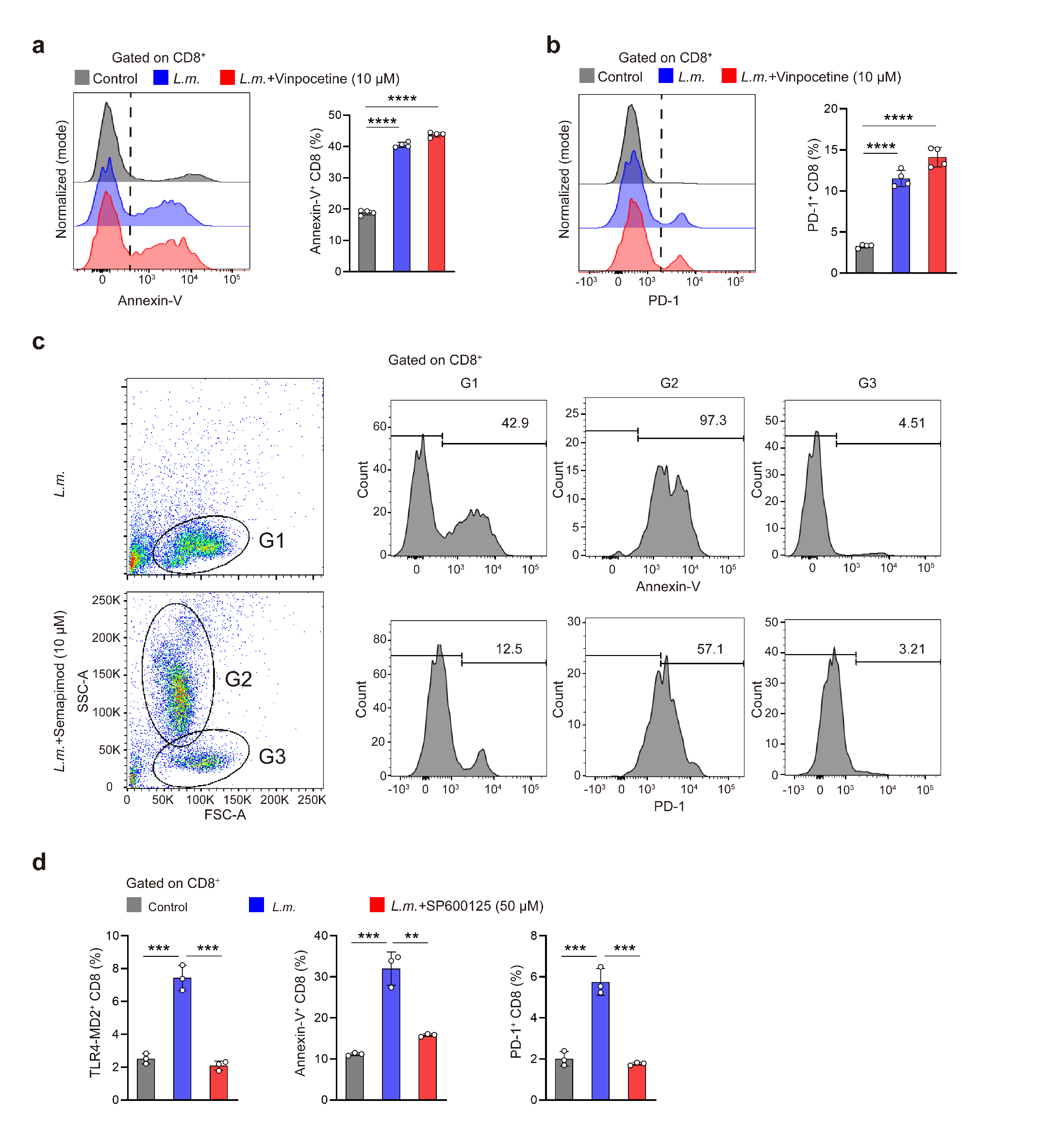


Figure. S6.

**Treatment with JNK inhibitors downregulates the apoptosis of CD8^+^ T cell upon *L.m.* infection. a**, **b** Representative FACS images and statistical analysis of Annexin-V^+^CD8^+^ (**a**) and PD-1^+^CD8^+^ (**b**) T cells with or without treatment of NF-κB inhibitor vinpocetine. **c** Representative FACS images of Annexin-V^+^CD8^+^ and PD-1^+^CD8^+^ T cells with or without treatment of P38 inhibitor semapimod. **d** Statistical analysis of TLR4-MD2^+^, Annexin-V^+^ and PD-1^+^ CD8^+^ T cells with JNK inhibitor SP600125 treatment after *L.m.* infection. Data are mean ± SD of one representative experiment. Similar results were seen in two or three independent experiments. Unpaired Student’s t tests. **p < 0.01, ***p < 0.001, ****p < 0.0001.


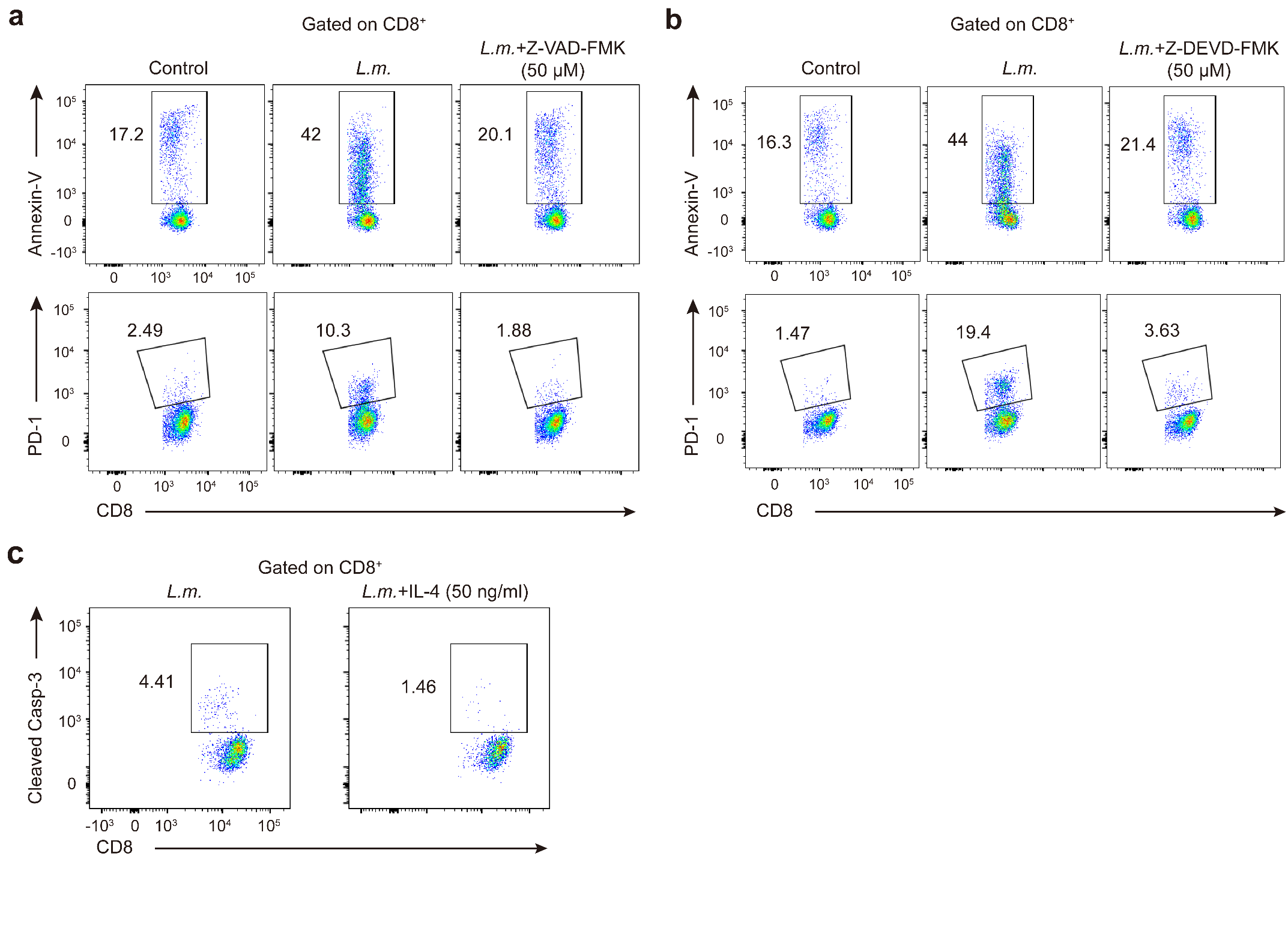


Figure. S7.

**IL-4 inhibits the Caspase-3-mediated CD8^+^ T cell apoptosis in response to *L.m.* infection. a**, **b** Representative dot plots of FACS analysis of Annexin-V^+^CD8^+^ and PD-1^+^CD8^+^ T cells with or without Z-VAD-FMK (**a**) and Z-DEVD-FMK (**b**) treatment after *L.m.* infection. **c** Representative dot plots of FACS analysis of cleaved caspase-3^+^CD8^+^T cells with or without IL-4 treatment after *L.m.* infection. Similar results were seen in two or three independent experiments.
